# Risks of Mycotoxins from Mycoinsecticides to Humans

**DOI:** 10.1155/2016/3194321

**Published:** 2016-04-10

**Authors:** Qiongbo Hu, Fuxia Li, Yuping Zhang

**Affiliations:** ^1^College of Agriculture, South China Agricultural University, Guangzhou 510642, China; ^2^Guangdong Provincial Key Laboratory of High Technology for Plant Protection, Plant Protection Research Institute, Guangdong Academy of Agricultural Sciences, Guangzhou 510640, China

## Abstract

There are more than thirty mycotoxins produced by fungal entomopathogens. Totally, they belong to two classes, NRP and PK mycotoxins. Most of mycotoxins have not been paid sufficient attention yet. Generally, mycotoxins do not exist in mycoinsecticide and might not be released to environments unless entomogenous fungus proliferates and produces mycotoxins in host insects or probably in plants. Some mycotoxins, destruxins as an example, are decomposed in host insects before they, with the insect's cadavers together, are released to environments. Many species of fungal entomopathogens have the endophytic characteristics. But we do not know if fungal entomopathogens produce mycotoxins in plants and release them to environments. On the contrary, the same mycotoxins produced by phytopathogens such as* Fusarium* spp. and* Aspergillus* spp. have been paid enough concerns. In conclusion, mycotoxins from mycoinsecticides have limited ways to enter environments. The risks of mycotoxins from mycoinsecticides contaminating foods are controllable.

## 1. Introduction

Entomopathogenic fungi are the important factors to control natural populations of many pest species. Several species have been developed as biological control agents (BCAs) from more than 800 species of fungal entomopathogens in the world. In the BCAs, there are more than 100 mycoinsecticides for commercial use worldwide [1]. And at least 30 mycoinsecticides were registered in China; among them,* Beauveria bassiana* is the most popular species up to 14 products for control of locust, pine moth and diamond back moth, and so forth;* Metarhizium anisopliae* and* Paecilomyces lilacinus* with the 8 and 7 products were registered to application of grubs, corn borer, aphids and whitefly, and so forth (http://www.chinapesticide.gov.cn/hysj/index.jhtml). There is much public interest in the use of fungal biological control agents as alternatives to chemical pesticides. However, there are some concerns about the safety of BCAs to human health. Many researches about the safety of BCAs have been carried on since the 21st century. Through assessing the risks of infections, allergies, and poisoning/toxic effects [2–4], the most used mycoinsecticides such as* B. bassiana* and* M. anisopliae* were verified as safe biocontrol agents [5–7]. However, many entomopathogens produce mycotoxins which pose risks to humans and the environment; how these mycotoxins affect human health and environment are not clear yet.

Numerous mycotoxins were found from fungal entomopathogens. They can be characterized to lots of classes according to the chemical structure [8]. But briefly, they can be classified as two main classes: nonribosomal peptide (NRP) synthetase mycotoxins and polyketide (PK) synthase mycotoxins according to their biosynthetic pathways.

## 2. NRP Mycotoxins

Fungal entomopathogens produce various kinds of NRPs that are usually taken as pathogenic factor of these fungi species. Chemically, NRPs are the secondary metabolic compounds mainly composed of specific or modified amino acids and hydroxyl acids. They are synthesized via thiotemplate multienzyme mechanism of multifunctional enzyme complex system other than on ribosome. NRP synthetase gene of fungi is an open reading frame encoding a peptide chain composed of several modules, which activate amino acids and combined with a specific peptide product. Each module has a number of domains, and a specific reaction is catalyzed by one domain. The main domains include adenylation domains (A domains), thiotion domains (T domains), condensation domains (C domains), epimerization domains (E domains), and methylation domains (M domains) [9].

**Figure 1:**
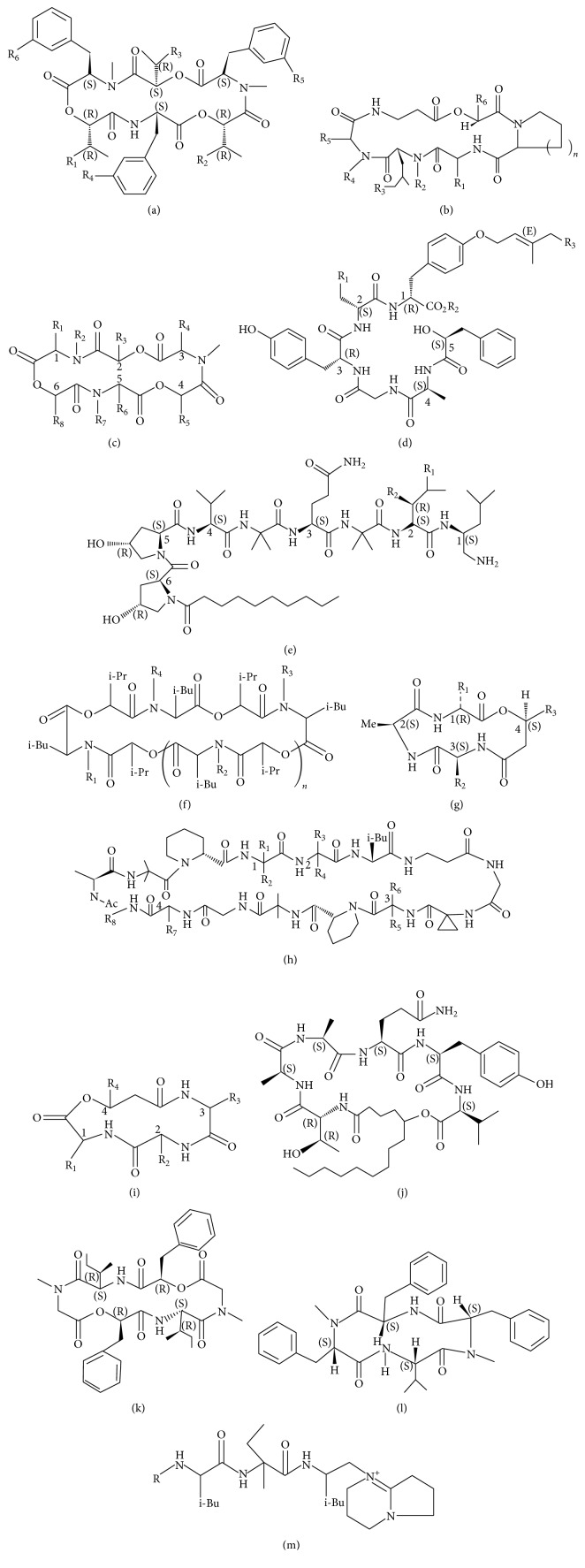
The structure of beauvericins (a), destruxins (b), enniatins (c), conoideocrellide (d), cicadapeptins (e), bassianolides (f), beauveriolides (g), neoefrapeptins (h), beauverolides (i), cordycommunin (j), hirsutellide (k), hirsutide (l), and efrapeptins (m).

**Figure 2:**
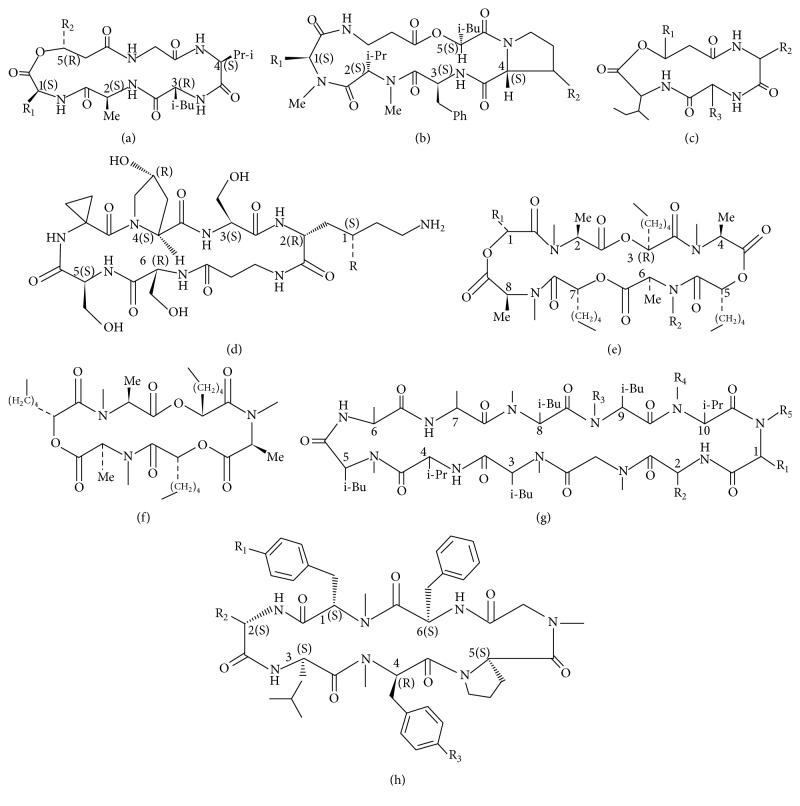
The structure of isariins (a), isaridins (b), isarolide (c), serinocyclins (d), verticilides A (e), verticilides B1 (f), cyclosporines (g), and cordyheptapeptide (h).

To date, more than twenty kinds of NRPs were isolated and identified from entomogenous fungi genera:* Beauveria, Conoideocrella, Cordyceps, Culicinomyces, Hirsutella, Isaria, Metarhizium, Paecilomyces, Verticillium*, and so forth. These NRPs include bassianolides, beauvericins, beauverolides, beauveriolides, cicadapeptins, conoideocrellides, cordycommunins, cordyheptapeptides, culicinins, cyclosporin, destruxins, efrapeptins, enniatins, hirsutellides, hirsutides, isariins, isaridins, isarolides, paecilodepsipeptides, and serinocyclins (Table 1, Figures 1 and 2). Every NRP above includes a series of analogues. Based on the molecular structures, the NRPs could be divided into chain peptides (e.g., cicadapeptin and efrapeptin) and cyclic peptides including a subdivision of cyclopeptides and cyclodepsipeptides. Cyclopeptides are cyclic structures built by amino acid residues through peptide bonding (e.g., cyclosporin), while cyclodepsipeptides are lactone compounds consisting of amino acids and hydroxyl acids which are connected by peptide bonds. Most of the NRPs belong to the group of cyclodepsipeptides [10]. To date, destruxins, beauvericins, and enniatins are the best researched NRPs. However, their detailed biosynthesis, biotransformation, and behavior and fate in the environments are not clear yet.

In all NRPs of entomogenous fungi, beauvericin is considered as emerging mycotoxins likely contaminating the foods and products including rice, wheat, maize, follow-up infant formula, and Chinese medicinal herbs [11–15]. The fungal entomopathogens of* Beauveria* spp.,* Paecilomyces* spp., and* Isaria* spp. produce beauvericins [16–18]. Traces of beauvericins were also detected in animal tissues and eggs [19, 20]. However, the cases of contaminations of beauvericins and enniatins are all from the infection of various* Fusarium* species other than entomogenous fungal species [15, 19, 21–25]. Chemically, beauvericins are a kind of cyclic hexadepsipeptide with alternating methyl-phenylalanyl and hydroxy-iso-valeryl residues (Figure 1(a)). Several documents reviewed beauvericins [15, 19, 21]. Totally 11 analogues of beauvericin were found [26]. The insecticidal effects of beauvericins at a microgram level were reported in several insects [21]. The cytotoxicity of beauvericins on human cells and cancer cells was also discovered [27–29]. Acetyl coenzyme-A (acyl-CoA: cholesterol acyltransferase, ACAT) is probably the target protein of beauvericins, while some research reports indicated that beauvericins might act as ionophores [30, 31].

Destruxins were isolated from culture medium of entomogenous fungii* M. anisopliae* and* Aschersonia* sp., and the fungal phytopathogen* Alternaria brassicicola* [32] (Figure 1(b)). Among 39 destruxin analogues, destruxins A, B, and E (DA, DB, and DE, resp.) are the most analogues and show substantial bioactivity [33]. However, the linear molecule resulting from the opening of the DA cycle is not toxic and DE would degrade to less toxic DE-diol upon enzymatic action [33]. Destruxins have insecticidal activity against many pests with various mode of action including contact action, gut toxicity, antifeedant effect, and ovicidal and oviposition deterrent activities [34]. Destruxins damage the innate immunity of insects [35–37]. Destruxin maybe acts as a kind of calcium ionophore and an inhibitor of V-H+-ATPase [38]. The antiviral, antitumor, and herbicidal activities and cytotoxicity were reported as well [33]. Destruxins were decomposed in host insects before they, with the cadaver, were released to environments, so it is unlikely to contaminate the food chains [39]. In fact, there are no records about residues of destruxins in agricultural products and foods.

Enniatins could be produced by the fungal entomopathogen,* Verticillium hemipterigenum* BCC 1449 [69]. Enniatins are N-methylated cyclohexadepsipeptides, composed of three units each of N-methylated branched-chain L-amino acid and D-2-hydroxy acid arranged in an alternate fashion (Figure 1(c)). To date, 29 enniatins have been isolated and characterized, either as a single compounds or as mixtures of inseparable analogues [70]. Enniatins have multiactivities including antifungal, antibiotic, and cytotoxic properties. Fusafungine, one drug developed from a mixture of enniatins, is used as a topical treatment of upper respiratory tract infections by oral and/or nasal inhalation. Enniatins inhibit ABC transporters [71]. Enniatins are also a common contaminant in grain-based foods, but they were produced by the fungal species of* Fusarium* spp. other than entomopathogens [11, 12, 72–74].

There is no information about other NRP mycotoxins influencing environments and human health.

## 3. PK Mycotoxins

**Figure 3:**
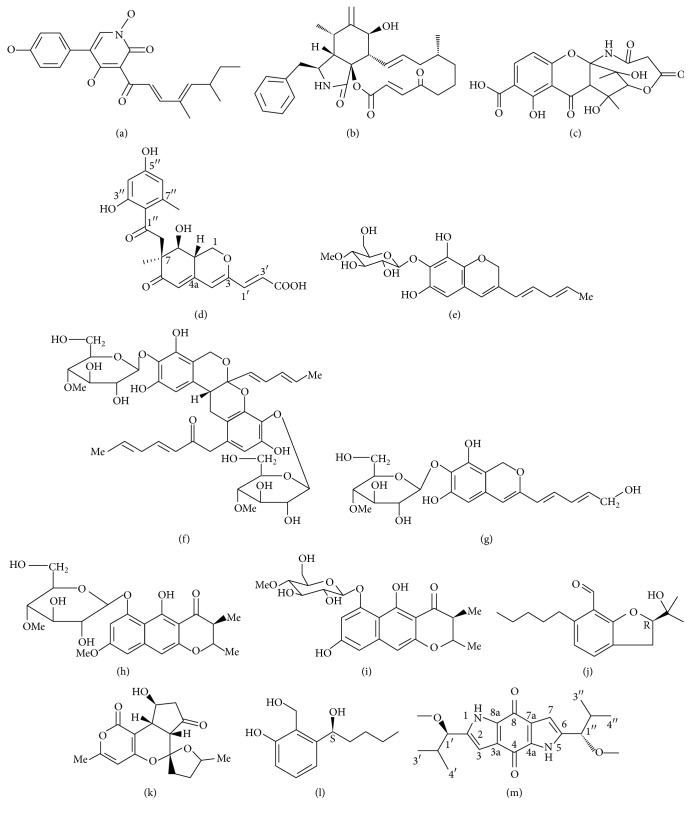
The structure of tenellin (a), cytochalasins (b), cryptosporioptide A (c), pinophilin C (d), indigotide A (e), indigotides C-F (f), 13-hydroxyindigotide A (g), 8-O-methylindigotide B (h), indigotide B (i), annullatin A (j), tenuipyrone (k), annullatin E (l), and terreusinone A (m).

**Figure 4:**
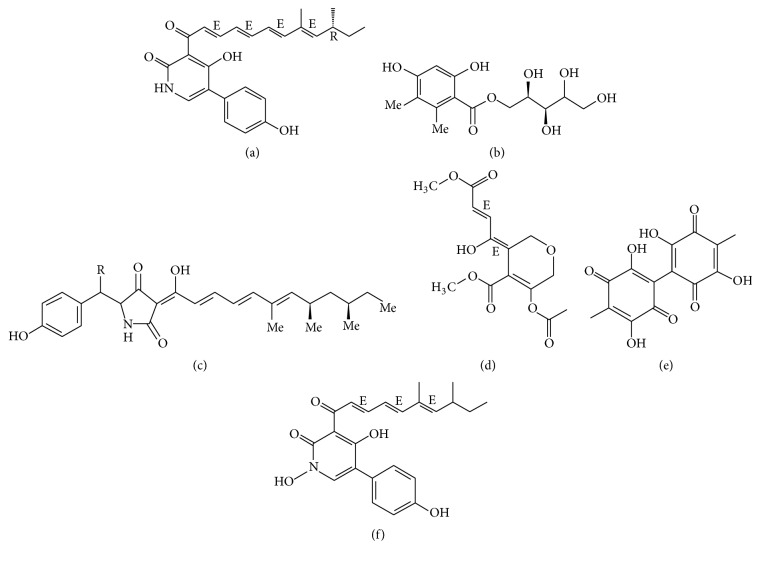
The structure of farinosones A (a), paeciloside A (b), and militarinones B (c), isariketide (d), oosporein (e), and bassianin (f).

Many fungal entomopathogen mycotoxins are polyketides and its derivatives (PKs); more than 20 PKs were discovered (Table 2, Figures 3 and 4). Fungal polyketide biosynthesis typically involves multiple enzymatic steps, and the encoding genes are often found in gene clusters. The enzymatic machinery for the formation of the polyketides consists of different modules characteristic of each fungus (e.g., keto synthases, acyl transferases, carboxylases, cyclases, dehydrases, aromatases, reductases, thioesterases, and laccases) [75].

One of the best characterised fungal polyketide synthesis pathways is that of the tenellin (Figure 3(a)) from the insect pathogen* B. bassiana* [76, 77]. Tenellin is not involved in insect pathogenesis [76], but tenellin acts as an iron chelator to prevent iron-generated reactive oxygen species toxicity in* B. bassiana* [78]. This toxin inhibits total erythrocyte membrane ATPase activity probably because of a consequence of membrane disruption, since all pigments caused alterations in erythrocyte morphology and promoted varying degrees of cell lysis [79]. There are no reports about the risk of tenellin as a mycotoxin to contaminate foods.

Oosporein (Figure 4(e)) is the major secondary metabolite excreted by* B. bassiana* [80] and* B. brongniartii* [81]. It had a median oral toxicity to 1-day-old cockerels [82]. Oosporein inhibits total erythrocyte membrane ATPase activity in a dose-dependent manner caused alterations in erythrocyte morphology and promoted varying degrees of cell lysis [79]; meanwhile, the toxin also exhibits broad spectrum of antimicrobial, antioxidant, and cytotoxic activities [83]. However, oosporein is a rather strong organic acid; it can be concluded that oosporein can hardly be adsorbed by organisms, so oosporein is unlikely to enter food chains and influence human health [81].

Bssianin (Figure 4(f)) is a PK pigment isolated from* B. bassiana*. It inhibits total erythrocyte membrane ATPase activity as well [79].

The fungal entomopathogen* M. anisopliae* produces cytochalasins (Figure 3(b)), a famous PK [84, 85]. Cytochalasins belong to a kind of cytochalasans which comprise diverse group of fungal polyketide-amino acid hybrid metabolites with a wide range of distinctive biological functions [86]. To date, more than 80 cytochalasans have been isolated from other fungi such as* Phomosis, Chalara, Hyposylon, Xylaria, Daldinia, Pseudeurotium,* and* Phoma exigua* [75]. Cytochalasans have phytotoxins or virulence factors and exhibit antimicrobial or cytotoxic activities and inhibit cholesterol synthesis or interfere with glucose transport and hormone release. However, the origin of their name is derived from the Greek terms kytos, meaning cell, and chalasis, meaning relaxation, pointing to the best known property of cytochalasans, the capping of actin filaments. As a result, cytokinesis is effectively inhibited while mitosis remains unaffected, thereby generating giant multinucleated or even, at higher concentrations, denucleated cells. These properties are exploited in molecular and cell biology research, especially in cell imaging methods, cytoskeleton, and cell cycle studies [86].

In the entomogenous fungal genus,* Cordyceps*, many species produce PKs. For example,* C. indigotica* produces aromatic polyketides, indigotides (Figure 3(f)), 13-hydroxyindigotide A (Figure 3(g)) and 8-O-methylindigotide B (Figure 3(h)) [91, 96]. Terreusinone A (Figure 3(m)), pinophilin C (Figure 3(d)), and cryptosporioptide A (Figure 3(c)) were isolated from* C. gracilioides*; these three compounds inhibit the activity of protein tyrosine phosphatases [88]. Annullatins (Figures 3(j) and 3(l)) were isolated from* C. annullata* [87]. Opaliferin, a polyketide with a unique partial structure in which a cyclopentanone and tetrahydrofuran were connected with an external double bond, was isolated from the insect pathogenic fungus* Cordyceps* sp. NBRC 106954 [93]. However, there is no information about the risks of these PK toxins to human health.

As to* Isaria* genus,* I. tenuipes* produces tenuipyrone (Figure 3(k)) [94].* I. felina* KMM 4639 produces isariketide Figure 4(d), showing moderate cytotoxicity toward HL-60 cells [99]. Militarinones were isolated from cultures of the* Cordyceps*-colonizing fungus* I. farinosa*. It showed significant cytotoxicity against A549 cells [100]. For the* Paecilomyces *genus, farinosones (Figure 4(a)) were isolated from the strain* Paecilomyces farinosus* RCEF 0101. They induce outgrowth but cytotoxicity in the PC-12 cell line [89]. Paeciloside A (Figure 4(b)) is isolated from* Paecilomyces* sp. CAFT156. Paeciloside A displays inhibitory effects on two gram-positive bacteria,* Bacillus subtilis* and* Staphylococcus aureus,* and moderate cytotoxicity towards brine shrimp larvae (*Artemia salina*) [101].* P. militaris* produces militarinones (Figure 4(c)) [92]. There is no other information of these PKs.

## 4. The Fate of Mycoinsecticide and Its Mycotoxins

In mycoinsecticide, the main component is usually the spores of fungal entomopathegen. Some of mycotoxins maybe exist inside of spores other than outside of spores. Mycoinsecticide itself is almost not the resource of mycotoxins. In fact, mycotoxins mainly come from the target pests or host insects infected by fungal entomopathogen of mycoinsecticide. The endophytic entomopathogenic fungus is maybe the other important mycotoxins resources. Of course, if considering the nonmycoinsecticide factors, the crops and products infected by other fungal species such as* Fusarium* spp.,* Aspergillus* spp. should be the more important resources of mycotoxins.

**Figure 5:**
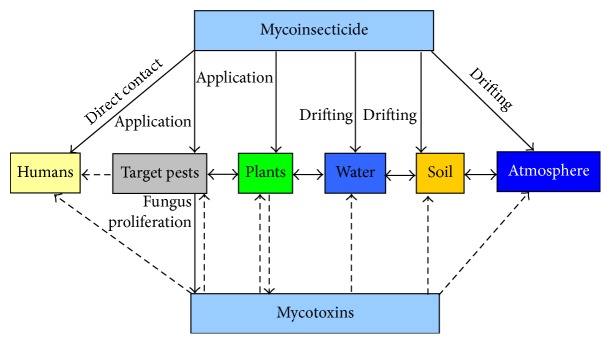
The fates of mycoinsecticide and its mycotoxins. → indicating the actually existing pathway, *⇢* indicating the pathway not found to date.

Totally, mycoinsecticide in its production and application has six fates (Figure 5). The first fate, humans, may be exposed to the risks of directly contacting the fungal entomopathogens. These humans are mainly the persons who long-timely produce and use the mycoinsecticide. There were several reports about fungal spores allergy of workers producing biocontrol agent of* Beauveria bassiana* and* Metarhizium anisopliae* [5, 6]. But there are no evidences supporting that the allergy is because of mycotoxins.

When mycoinsecticide is used, the important fate is the target insects. The fungal spores of mycoinsecticide adhere insect surface and then start a pathogenic progress. After penetrating the cuticle, the fungus proliferates itself and produces mycotoxins in host insect. At last, the fungal phages and its mycotoxins along with the cadavers of host insects are released to environments. To date, we do not know how many of the mycotoxins enter the environment. However, a few research cases indicate that the mycotoxins from entomopathogens are scarcely released to environments. For example, the amount and type of destruxin produced are dependent upon the fungal strain and insect host and the fact that these compounds decomposed shortly after host death. Destruxin decomposition was presumably due to the activity of hydrolytic enzymes in the cadavers and appeared to be independent of host or soil type and biota. So, destruxins are essentially restricted to the host and pathogen and are unlikely to contaminate the environment or enter the food chain [39].

Plants including target crop and weeds are the important fate of mycoinsecticide (Figure 5). The main fungal resources of plants is from mycoinsecticide application and target pests. Plants maybe hardly receive the fungus from the systems of water, soil, and atmosphere. Fungal entomopathogen is not phytopathogen, and in general, the phages of fungal entomopathogen only deposit the plants surface. However, many species of entomogenous fungi such as* B. bassiana, M. anisopliae*, and* I. fumosorosea* have been found the endophytic characteristics [102–104]. If so, the detection and management of mycotoxins from fungal entomopathogens are becoming more important, especially for those food crops.

Soil is an important storage bank of fungal entomopathogens. Fungal spores in soil can survive for long time. Through drifting from application and dropping from target pests cadavers, fungal phages and mycotoxins maybe enter the soil system.* Beauveria* spp.,* Metarhizium* spp.,* Paecilomyces* spp., and* Isaria* spp. can be often isolated from soil [105] and the entomopathogens in soil can be detected after mycoinsecticide is used [106]. But there are no reports that mycotoxins of fungal entomopathogen are detected in soil.

Water is another fate of mycoinsecticide. Beauvericins were detected in drainage water after* Fusarium* spp. was inoculated on wheat plants [107]. However, there are no researches indicating mycotoxins from mycoinsecticides entering the water system.

Atmosphere obtains fungal entomopathogens from drifting. Also, fungus might be exchanged between soil, water, and atmosphere systems. But we can not ensure that fungal mycotoxins enter atmosphere.

## 5. Conclusion

There are more than thirty mycotoxins isolated from fungal entomopathogens. Based on the biosynthesis, they are classified to NRP and PK mycotoxins. Beauvericins, enniatins, destruxins, cytochalasins, and tenellin are given relevantly intensive researches; other mycotoxins have not been paid sufficient attention. Mycotoxins are produced by cells of fungal entomopathogens used as mycoinsecticide. But mycotoxins are generally not in mycoinsecticide. So, mycotoxins might not be released to environments unless fungus proliferates and produces mycotoxins in host insects or probably in plants. To date, we only know little information about if mycotoxins enter environments. For example, destruxins were decomposed in host insects before they, with the cadaver, were released to environments [39]. Although entomopathogenic fungi are generally not the plants pathogens, many of them have the endophytic characteristics. However, we nowadays neither know if fungal entomopathogens produce mycotoxins in plants and release them to environments nor have enough information that the food chains are contaminated by mycotoxins the host insect produced and that human health are influenced by them. On the contrary, the same mycotoxins produced by phytopathogens such as* Fusarium* spp.,* Aspergillus* spp. have been paid more attention.

In conclusion, mycotoxins from mycoinsecticides have limited ways to enter environments. The risks of mycotoxins from mycoinsecticides contaminating foods are likely controllable.

## Figures and Tables

**Table 1:** NRP mycotoxins of fugal entomopathogens.

Mycotoxin name	Producing fungal entomopathogen	Bioactivity	References
Cicadapeptins	*Cordyceps heteropoda* *Isaria sinclairii*	Inhibits acetylcholine- (Ach-) evoked secretion Antibacterial activity	[40–42]

Culicinins	*Culicinomyces clavisporus*	Inhibits breast tumor cells	[43, 44]

Efrapeptins	*Tolypocladium *spp.	Insecticidal activity; anti-immunity; antifungal activities; inhibitors of F1F0-ATPase	[45, 46]

Neoefrapeptins	*Geotrichum candidum*	Insecticidal activities	[47]

Bassianolides	*Beauveria bassiana* *Verticillium lecanii*	Inhibit muscle contraction Insecticidal activities	[48]

Beauvericins	*Beauveria *spp. *Paecilomyces *spp.	Insecticidal Fungicidal activity Cytotoxic Antitumor	[28, 49–51]

Beauverolides	*Beauveria *spp. *Paecilomyces *spp.	Inhibits insect immunity	[26]

Beauveriolides	*Beauveria *spp.	Antiatherogenic Antiobesity activities	[26]

Conoideocrellide	*Conoideocrella tenuis* *Paecilomyces militaris*		[52]

Paecilodepsipeptides	*Paecilomyces cinnamomeus*	Antiproliferative activity Antitumor activity	[52–55]

Cordycommunin	*Ophiocordyceps communis*	Inhibits *Mycobacterium tuberculosis*	[56]

Destruxins	*Metarhizium anisopliae* *Aschersonia *sp.	Insecticidal Herbicidal Cytotoxic	[26, 32, 33, 57]

Hirsutellides	*Hirsutella kobayasii*	Antimalarial	[58]

Hirsutides	*Hirsutella* spp.		[59]

Isariins	*Isaria cretacea*	Insecticidal activity	[26, 60]

Isaridins	*Isaria* spp.	Inhibits growth of *Plasmodium falciparum*	[26, 61, 62]

Isarolides	*Isaria *spp.		[26]

Serinocyclins	*Metarhizium anisopliae*	Insecticidal activity	[63]

Verticilides	*Verticillium *spp.	Inhibits acyl-CoA: cholesterol acyltransferase of CHO cells Inhibits ryanodine receptors of cockroach	[64–66]

Cyclosporines	*Beauveria* *Verticillium* *Trichoderma polysporum* *Cylindrocarpon lucidum*	Insecticidal activities Immunosuppressive effect	[26]

Cordyheptapeptides	*Cordyceps* spp.	Antimalarial activity Cytotoxicity to Vero cell lines	[67, 68]

**Table 2:** PK mycotoxins of fugal entomopathogens.

Mycotoxin name	Producing fungal entomopathogen	Bioactivity	References
Annullatins	*Cordyceps annullata*	Exhibit potent agonistic activity toward the cannabinoid receptors CB1 and CB2.	[87]

Cryptosporioptide A	*Cordyceps gracilioides*	Inhibit the activity of protein tyrosine phosphatases	[88]

Cytochalasins	*Metarhizium anisopliae*	Inhibitor of the actin-cofilin interaction	[84, 85]

Farinosones A, B, and C	*Paecilomyces farinosus*	Cytotoxic	[89]

Fumosorinones	*Isaria fumosorosea*	Inhibits tyrosine phosphatase 1B (PTP1B) to treat type 2 diabetes mellitus (T2DM)	[90]

Indigotides	*Cordyceps indigotica*		[91]

Militarinones	*Paecilomyces militaris*	Cytotoxic	[92]

Opaliferin	*Cordyceps* sp.		[93]

Pinophilin C	*Cordyceps gracilioides*	Inhibit the activity of protein tyrosine phosphatases	[88]

Tenellin	*Beauveria bassiana*		[76, 78]

Terreusinone A	*Cordyceps gracilioides*	Inhibit the activity of protein tyrosine phosphatases	[88]

Tenuipyrone	*Isaria tenuipes*		[94]

Torrubiellones	*Torrubiella *sp.	Antimalarial	[95]

13-Hydroxyindigotide A	*Cordyceps indigotica*		[96]

8-O-Methylindigotide B	*Cordyceps indigotica*		[96]

Oosporein	*Cordyceps cardinalis*	Antibiotic Antifungal Antitumor	[83, 97]

Bassianin	*Beauveria *spp.	Inhibits erythrocyte membrane ATPase and inhibits Ca^2+^-ATPases	[98]
